# Isolation and Identification of Two New Polyynes from a North American Ethnic Medicinal Plant--*Oplopanax horridus* (Smith) Miq

**DOI:** 10.3390/molecules15021089

**Published:** 2010-02-23

**Authors:** Wei-Hua Huang, Qing-Wen Zhang, Chong-Zhi Wang, Chun-Su Yuan, Shao-Ping Li

**Affiliations:** 1 Institute of Chinese Medical Sciences, University of Macau, Macao, China; E-Mail:endeavor34852@yahoo.com.cn (W.-H.H.); 2 Tang Center for Herbal Medicine Research, The Pritzker School of Medicine, University of Chicago, Chicago, IL 60637, USA; E-Mails: czwang@dacc.uchicago.edu (C.-Z.W.); CYuan@dacc.uchicago.edu (C.-S.Y.)

**Keywords:** *Oplopanax horridus*, polyyne, oplopantriol A and B, alkaline hydrolysis

## Abstract

Two new polyynes, named oplopantriol A (**5**) and oplopantriol B (**6**), were isolated from the root bark of *Oplopanax horridus* (Smith) Miq, an ethnic medicinal plant of North America, along with four known polyynes: (3*S*,8*S*)-falcarindiol (**1**), oplopandiol (**2**), (11*S*,16*S*,9*Z*)-9,17-octadecadiene-12,14-diyne-1,11,16-triol, 1-acetate (**3**) and oplopandiol acetate (**4**). The structures of the new compounds were elucidated by detailed spectroscopic analyses, including 1D and 2D NMR techniques and chemical methods. The absolute configurations of the new compounds **5** and **6** were determined by comparing their optical rotation values with the hydrolysis products of the known compounds **3** and **4**, respectively, derived from the same plant. On the basis of an analysis of their physical and chemical properties we show that the alkaline hydrolysis of **3** and **4 **afforded the new compounds **5** and **6**, respectively.

## 1. Introduction

**Figure 1:**
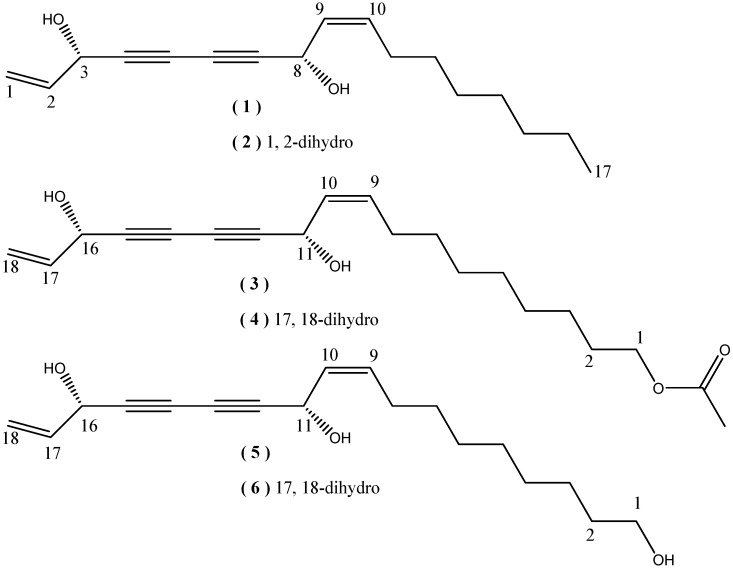
Structures of compounds **1**-**6**.

Plants of the genus *Oplopanax*, belonging to the family Araliaceae, comprise three species which are *Oplopanax japonicus* (Nakai) Nakai. uniquely found in Japan, *Oplopanax elatus* Nakai, only distributed in northeast China, and *Oplopanax horridus* (Smith) Miq. exclusively originated and grown in North America [1,2,3]. These ethnic medicinal herbs were reported to have anti-tuberculosis, antibiotic, lineae atrophicae relieving, antifungal, anti-psoriasis and anticancer activities [4,5,6,7,8,9]. *O. horridus*, commonly known as Devil’s Club, whose inner bark and roots are used by First Nations peoples for a variety of ailments such as diabetes, rheumatism, tuberculosis, colds, headaches, and lung hemorrhages [10], was reported to afford antimycobacterial and antifungal polyyne ingredients [11]. As a part of our research work on bioactive metabolites from the plants of *Oplopanax*, phytochemical investigation on *O. horridus* was conducted and two new polyynes **5** and **6** were isolated, together with four known polyynes: (3*S*,8*S*)-falcarindiol (**1**), oplopandiol (**2**), (11*S*,16*S*,9*Z*)-9,17-octadecadiene-12,14-diyne- 1,11,16 -triol, 1-acetate (**3**) and oplopandiol acetate (**4**) (Figure 1) [11]. Although the planar structure of compound **5** was reported previously, the absolute configuration was not elucidated [12]. The present paper describes the isolation and structural elucidation of compounds **5** and **6** on the basis of the IR, ^1^H- and ^13^C-NMR, Hydrogen-Hydrogen Correlation Spectroscopy (H-H COSY), Heteronuclear Multiple Quantum Coherence (HMQC), Heteronuclear Multiple Bond Coherence (HMBC), mass spectroscopic data and chemical methods.

## 2. Results and Discussion

By successive column chromatography (CC) on silica gel and octadecyl silica gel (ODS gel) and prep-HPLC, an 85% ethanol extract of air-dried root bark of *O. horridus* afforded two new polyynes **5**, **6**, along with four known compounds **1**-**4**. The identification of **5** and **6** were made by spectroscopic data. The absolute configurations of the new compounds **5** and **6** were determined by comparing their optical rotation values with the hydrolysis products of the known compounds **3** and **4, **respectively, derived from the same plant. 

**Figure 2:**
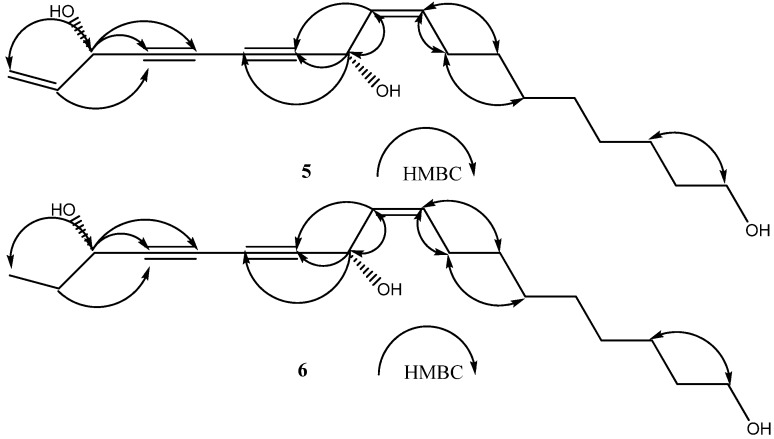
Key HMBC correlations (H → C) of compounds **5** and **6**.

Compound **5** was obtained as a yellowish oil. The molecular formula of **5** was determined to be C_18_H_26_O_3_ on the basis of the HR-electrospray ionization (ESI)-MS spectrum (*m/z* 289.1867 [M-H]¯, Calcd for C_18_H_25_O_3_: 289.1804). The UV (288, 271, 263, and 253 nm) and IR absorptions (2250 and 1675 cm^–1^) indicated the presence of two C≡C bonds [13]. The ^1^H-NMR spectrum of **5** displayed signals due to five olefinic protons at *δ_H _*5.93 (ddd, *J* = 17.4, 10.0, 5.5 Hz), 5.58 (ddt, *J* = 10.6, 7.3, 1.0 Hz), 5.51 (ddt, *J* = 10.6, 8.2, 1.0 Hz), 5.46 (dt, *J* = 17.4, 1.0 Hz) and 5.22 (dt, *J* = 10.0, 1.0 Hz), in addition to a hydroxymethyl group at *δ_H_* 3.64 (2H, t, *J* = 6.5 Hz), seven methylene groups at *δ_H_* 2.11 (2H, tq, *J* = 7.1, 1.5 Hz), 1.56 (2H, m), 1.39 (2H, m) and 1.31 (8H, m) (Table 1). Analysis of the ^13^C-NMR and HMQC spectra revealed the presence of 18 carbons (Table 1), containing seven methylenes carbons (*δ_C_* 25.6-32.6) and one hydroxymethyl at *δ_C_* 63.0, four olefinic carbons at *δ_C_* 136.0, 134.2, 127.9 and 117.1, four unprotonated acetylenic carbons and two oxygen-bearing sp^3 ^carbons at *δ_C_* 58.5 and 63.3. All protonated C-atoms and their corresponding H-atoms were assigned by the HMQC experiments. The structure elucidation was assisted by analyses of the HMBC experiments (Figure 2). The HMBC correlations between H-16 (*δ_H_* 4.93) and C-18 (*δ_C_* 117.1), C-17 (*δ_C_* 136.0), C-15 (*δ_C_* 78.5) and C-14 (*δ_C_* 70.1) indicated that the hydroxy group was connected to C-16. Furthermore, the correlations between H-11 (*δ_H_* 5.19) and C-13 (*δ_C_* 68.7), C-12 (*δ_C_* 79.8), C-9 (*δ_C_* 127.9) and C-10 (*δ_C_* 134.2) identified that another hydroxyl group was attached to C-11. The correlations in the H-H COSY spectrum between the hydroxy methylene at *δ_H_* 5.19 and olefinic proton at *δ_H_* 5.58 as well as between the other hydroxy methylene at *δ_H_* 4.93 and another olefinic proton at *δ_H_* 5.93 confirmed above findings. The geometry of the double bond between C-9 and C-10 was determined to be *cis* as the alkene bond was fixed to be ***Z*** according to the vicinal coupling constant between H-9 and H-10 (*J_9,10_* = 10.6 Hz). On the basis of these structural determinations, the planar structure of **5** was established as 9,17-octadecadien-12,14- diyne-1,11,16-triol. The absolute configuration of compound **5** was not elucidated, but would be determined together with that of compound **6**. 

Compound **6** was prepared as yellowish oil. Its molecular formula, C_18_H_28_O_3_, was determined from the [M-H]¯ peak at 291.1966 (Calcd for C_18_H_27_O_3_: 291.1960), in the HR-ESI-MS spectrum. The UV (287, 261, 255, and 226 nm) and IR absorptions (2232 and 1656 cm^–1^) suggested that compound **6** had the same structural skeleton as compound **5**. The ^1^H and ^13^C NMR (Table 1) spectra of **6** were similar to those of **5**, except for the appearance of signals for an ethyl group [*δ*_H_ 1.00 (3H, t, *J* = 7.5 Hz) and 1.74 (2H, m); *δ*_C_ 9.3 and 30.6] and the disappearance of the signals for the terminal double bond [*δ*_H_ 5.52 (1H, dt, *J* = 10.0 and 1.0 Hz), 5.46(3H, dt, *J* = 17.6 and 1.0 Hz) and 5.93 (3H, ddd, *J* = 17.4, 10.0 and 5.5 Hz); *δ*_C_ 117.1 and 136.0], suggesting that **6** was a dihydro derivative of **5**. In the HMBC spectrum (Figure 2), the correlations between H-16 [*δ*_H_ 4.37(1H, t, *J* = 6.6 Hz)] and C-17 (*δ*_C_ 30.6), C-18 (*δ*_C_ 9.3), C-15 (*δ*_C_ 80.9) and C-14 (*δ*_C_ 68.8), as well as between H-11 [*δ*_H_ 5.19 (1H, br.d, *J* = 8.2 Hz)] and C-10 (*δ*_C_ 134.1), C-9 (*δ*_C_ 128.0), C-12 (*δ*_C_ 79.1) and C-13 (*δ*_C_ 68.8) confirmed the structure as shown in Figure 1. The geometry of the double bond between C-10 and C-9 was determined to be the same as compound **5** as *cis* and ***Z***. According to the above results, the planar structure of **6** was elucidated to be 9-octadecaen-12,14-diyne-1,11,16-triol.

**Table 1:** ^1^H- (500 MHz) and ^13^C-NMR (125 MHz) data of **5** and **6** in CDCl_3_^α, β^.

Carbon position	compound 5			compound 6	
	*δ*_H_ (*J* in Hz)	*δ*_C_		*δ*_H_ (*J* in Hz)	*δ*_C_
1	3.64, t (2H, 6.5 )	63.0		3.64, t (2H, 6.5 )	63.0
2	1.56, m (2H )	32.6		1.57, m ( 2H )	32.6
3	1.31, m (2H)	25.6		1.31, m (2H)	25.6
4	1.31, m (2H)	29.1 *^a^*		1.31, m (2H)	29.1 *^b^*
5	1.31, m (2H)	29.2		1.31, m (2H)	29.2
6	1.31, m (2H)	29.0 *^ a^*		1.31, m (2H)	29.0 *^ b^*
7	1.39, m (2H)	28.8		1.38, m (2H)	28.8
8	2.11, dq (2H, 7.1, 1.5 )	27.5		2.11, dq (2H, 7.1, 1.5 )	27.5
9	5.51, ddt (1H, 10.6, 8.2, 1.5 )	127.9		5.52, ddt (1H, 10.6, 8.2, 1.5 )	128.0
10	5.58, ddt (1H, 10.6, 7.3, 1.5 )	134.2		5.58, ddt (1H, 10.6, 7.3, 1.5 )	134.1
11	5.19, d (1H, 8.0)	58.5		5.19, br.d (1H, 8.0)	58.5
12	-	79.8		-	79.1
13	-	68.7		-	68.8 *^c^*
14	-	70.1		-	68.8 *^ c^*
15	-	78.5		-	80.9
16	4.93, br.d ( 1H, 5.5)	63.3		4.37, t ( 1H, 6.6)	63.8
17	5.93, ddd ( 1H, 17.4, 10.0, 5.5 )	136.0		1.74, m (2H)	30.6
18	5.22, dt ( 1H, 10.0, 1.0 );5.46, dt ( 1H, 17.4, 1.0 )	117.1		1.00, t ( 3H, 7.5)	9.3

^α^ TMS was used as an internal standard in spectra experiments; ^β^ Assignments based on HMQC and HMBC experiments; *^a-c^* Assignments may be interchanged.

Alkaline hydrolysis of **3 **and **4** afforded their deacetyl derivatives **3a** and **4a**, respectively, which had the same retention times as **5 **and **6** by Ultra Performance Liquid Chromatography (UPLC) analysis. Furthermore, the optical rotation values of **3a** {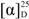 + 189.3° (*c* = 0.23, CHCl_3_)} and **4a** {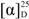 + 230.6° (*c* = 0.11, CHCl_3_)} were identical with those of the new polyynes **5** {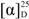 +194.4° (*c* = 0.16, CHCl_3_)}and **6**{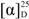 + 233.0° (*c* = 0.3, CHCl_3_)}, respectively. The above evidence indicated that **5** and **6** should have the same absolute configurations with the known compounds **3** and**4**. Thus, the complete structures of the new polyynes, oplopantriol A (**5**) and oplopantriol B (**6**), were elucidated to be (11*S*,16*S*,9*Z*)-9,17-octadecadien-12,14-diyne-1,11,16-triol and (11*S*,16*S*,9*Z*)-9-octadecaen-12,14- diyne-1,11,16-triol, which were named as oplopantriol A (**5**) and oplopantriol B (**6**), respectively.

Falcarindiol was isolated from several species in Araliaceae, Asteraceae and Apiaceae. The absolute configuration of falcarindiol from *Peucedanum oreoselinum* was assigned as (3*R*,8*S*) by Lemmich in 1981 on the basis of chemical correlation studies [14], and the same result was obtained by Ratnayake and Hemscheidt using olefin cross-metathesis for that isolated from *Tetraplasandra hawaiiensis* [15]. Steroselective synthesis of (3*R*,8*S*)-falcarindiol has been reported by Zheng *et al*. [16] and Sabitha *et al*. [17]. The (3*S*,8*S*) epimer was also reported by Bernart *et al*. and Kobaisy *et al*. from *Dendropanax arboreus* [18] and *O. horridus* [11], respectively. In Mosher’s method, the resonances of falcarindiol with a (3*R*,8*S*)-configuration for protons H-9, H-10, and H-11 all showed negative Δ*δ* (*δS* – *δR*)values, and those of the resonances for H-1*E*, H-1*Z*, and H-2 were all positive (the data were extracted from the supporting materials of reference [15], and was misinterpreted in the text), while that with a (3*S*,8*S*)-configuration had shown all negative Δ*δ* values [18]. The stereochemistry found for polyynes isolated from Araliaceae with a (3*S*,8*S*)-configuration seems to be entirely different from those with the (3*R*,8*S*) stereochemistry reported from Apiaceae and Asteraceae [14,19,20,21,22]. Consequently the four known polyynes were proposed as (3*S*,8*S*)-falcarindiol (**1**), oplopandiol (**2**), (11*S*,16*S*,9*Z*)- 9,17-octadecadiene-12,14-diyne-1,11,16-triol, 1-acetate (**3**) and oplopandiol acetate (**4**) with a (3*S*,8*S*)-configuration or (11*S*,16*S*)-configuration on basis of biosynthesis pathway, optical rotation values and spectroscopic data with those reported from the same plants.

## 3. Experimental

### 3.1. General

Optical rotations were measured on a PerkinElmer Model 341 polarimeter. UV spectra were recorded on a Beckman Coulter DU 640 spectrophotometer. IR spectra were obtained with a PerkinElmer Spectrum 100 FT-IR spectrometer with KBr pallets. The ^1^H-, ^13^C-, and 2D-NMR spectra were recorded on a Bruker AV-500 spectrometer at room temperature (*δ* in ppm, *J* in Hz) with tetramethylsilane (TMS) as an internal standard (Bruker, Germany). ESI-MS and HR-ESI-MS measurements were carried out on an Agilent 1100 series LC/MSD Trap VL mass spectrometer and a Wiff Agilent time-of-flight (TOF) mass spectrometer respectively (Agilent, USA). Silica gel (100-200 and 200-300 mesh) (Qingdao Haiyang Chemical Co. Ltd, China) and Alltech Reversed-phase C_18_ (RP-C_18_) silica gel (40-63*µ*m) (Alltech, USA) were used for column chromatography (CC). Precoated silica gel GF_254_ plates (Qingdao Haiyang Chemical Co. Ltd, Qingdao, China) were used for TLC. Supercritical fluid extraction was manipulated on a supercritical fluid extractor (SFT-250, Supercritical Fluid Technologies, Inc., USA). Analytical HPLC was performed on an Agilent 1100 liquid chromatograph with an Alltech Alltima RP-C_18_ column (250 mm × 4.6 mm inside diameter (I.D.), 5 *µ*m, Alltech, USA). Preparative HPLC was carried out with an Agilent 1100 liquid chromatograph with an Alltech Alltima RP-C_18_ column (250 mm × 22 mm I.D., 10 *µ*m). Analytical UPLC was performed on Waters Acquity Ultra performance LC (Waters, Milford, MA), equipped with binary solvent manager, sampler manager, column compartment, and PDA detector, connected to Waters Empower 2 software, with an Acquity UPLC BEH C_18_ column (50 mm × 2.1 mm I.D., 1.7 μm). HPLC-grade methanol was a product of Merck (Merck, Germany). The deionized water used for HPLC was purified by a Milli-Q purification system (Millipore, USA).

### 3.2. Plant Material

The dried root bark of *O. horridus* was collected and authenticated by one of the authors (C.-Z. Wang) from Chicago, IL of USA in March, 2009. A voucher specimen has been deposited in the Laboratory of Quality Control, Institute of Chinese Medicine Sciences, University of Macau, Macao, China. 

### 3.3. Extraction and Isolation

After the volatile oil was removed from the air-dried, powdered root bark of *O.* *horridus* (10.5 kg) by supercritical fluid extraction (SFE), the residue (10.2 kg) was extracted by 85% EtOH under refluxing, and the crude extract (1,900 g) was suspended in water and then extracted successively with petroleum ether (60-90°C), EtOAc, and *n*-BuOH to give the corresponding fractions P (124 g), E (570 g) and B (610 g), respectively. The EtOAc-soluble fraction E (510 g) was separated by silica gel (100–200 mesh) CC, eluted with a gradient of CHCl_3_–MeOH (50:1 to 0:1) to give ten fractions (E1–E10). Fraction E7 (82 g) was then subjected to CC of silica gel (200–300 mesh), eluting with CHCl_3_–MeOH(10:1, 8:1 and 5:1), to give six subfractions (E7a–E7f). Subfraction E7d (50 g) was chromatographed on RP-C_18_ silica gel CC (MeOH-H_2_O, 70:30), then prepared on Prep-HPLC (MeOH-H_2_O, 78:22) to afford **1** (1.6g) and **2** (2.5 g). Fraction E8 (75 g) was subjected to silica gel (200–300 mesh) CC, eluting with CHCl_3_–MeOH(10:1, 6:1 and 4:1), to afford five subfractions (E8a–E8e). Subfraction E8d (45 g) was chromatographed on RP-C_18_ silica gel CC (MeOH-H_2_O, 67:33), then by prep-HPLC (MeOH-H_2_O, 70:30) to afford **3** (2.6 g) and **4** (3.0 g). Subfraction E9 (68 g) was further separated by CC on silica gel (200–300 mesh), eluting with CHCl_3_–MeOH(8:1, 5:1 and 3:1), to yield six subfractions (E9a–E9f). Subfraction E9e (36 g) was further purified by prep-HPLC (MeOH-H_2_O, 65:35) to afford **5** (1.8 g) and **6** (2.1 g).

### 3.4. Alkaline Hydrolysis of Compounds ***3*** and ***4***

The polyyne ester compound **3** (21 mg) and compound **4** (22 mg) were each dissolved in 95% ethanol (1 mL). Then, NaOH (8 mg) was added to each solution, and the mixtures were heated at 60 °C for 4 hours. The mixtures were diluted with H_2_O (5 mL) and each one was extracted with CHCl_3_ (6 mL × 3). The CHCl_3_ layer was evaporated and the hydrolysis products were subjected to prep-HPLC (MeOH-H_2_O, 65:35) to afford **5** (6 mg) and **6** (5 mg), respectively.

*Oplopantriol* **A** (**5**), (11*S*,16*S*,9*Z*)-9,17-octadecadien-12,14-diyne-1,11,16-triol yellowish oil; 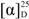 +194.4° (*c* = 0.16, CHCl_3_); UV (CHCl_3_) λmax (log ξ): 215 (0.63), 226 (1.10), 255 (4.09) 261 (3.95), 273 (4.13) and 287 (4.07) nm; IR (KBr) ν_max_ : 3357, 3022, 2929, 2855, 2251, 2150, 1675, 1464, 1405, 1303, 1021, 933 and 880 cm^-1^; ^1^H and ^13^C NMR data (Table 1); Positive mode ESI-MS *m/z*: 313 [M+Na]^+^ (100); Negative mode HR-ESI-MS *m/z*: 289.1867 [M-H]¯, Calcd for C_18_H_25_O_3_: 289.1804).

*Oplopantriol* **B** (**6**), (11*S*,16*S*,9*Z*)-9-octadecaen-12,14-diyne-1,11,16-triol yellowish oil; 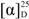 + 233.0° (*c* = 0.3, CHCl_3_); UV(CHCl_3_) λmax (log ξ): 207 (1.07), 226 (1.19), 232 (1.17), 253 (4.02), 263 (3.98), 265 (3.95), 272 (4.01) and 288 (3.84) nm; IR (KBr) ν_max_ : 3355, 3021, 2930, 2856, 2232, 2143, 1656, 1463, 1305, 1095, 1017, 970 and 866 cm^-1^; ^1^H and ^13^C NMR data (Table 1); Positive mode ESI-MS *m/z*: 315 [M+Na]^+^ (100); Negative mode HR-ESI-MS *m/z*: 291.1966 [M-H]¯, Calcd for C_18_H_27_O_3_: 291.1960).

## 4. Conclusions

A detailed phytochemical investigation on *O. horridus* led to the isolation of two new polyynes (11*S*,16*S*,9*Z*)-9,17-Octadecadien-12,14-diyne-1,11,16-triol and (11*S*,16*S*,9*Z*)-9-octadecaen-12,14- diyne-1,11,16-triol named oplopantriol A (**5**) and oplopantriol B (**6**), along with four known polyynes (3*S*,8*S*)-falcarindiol (**1**), oplopandiol (**2**), (11*S*,16*S*,9*Z*)-9,17-octadecadiene-12,14-diyne-1,11,16-triol, 1-acetate (**3**) and oplopandiol acetate (**4**). 
